# Dynamic Upper and Lower Airway Microbiotas in Paediatric Bronchiectasis Exacerbations: A Pilot Study

**DOI:** 10.3389/fcimb.2021.773496

**Published:** 2022-01-24

**Authors:** David T. J. Broderick, Tyler Regtien, Alana Ainsworth, Michael W. Taylor, Naveen Pillarisetti

**Affiliations:** ^1^ School of Biological Sciences, University of Auckland, Auckland, New Zealand; ^2^ Department of Paediatric Respiratory Medicine, Starship Children’s Hospital, Auckland, New Zealand

**Keywords:** microbiota (16S), bronchiectasis, airway microbiota, paediatric, exacerbation

## Abstract

**Introduction:**

Non-cystic fibrosis bronchiectasis is a respiratory health condition with many possible aetiologies, some of which are potentially reversible in childhood with early diagnosis and appropriate treatment. It is important to understand factors which contribute to progression or potential resolution of bronchiectasis. It is evident that respiratory exacerbations are a key feature of bronchiectasis disease progression. In this pilot study we document how the microbiota of the upper and lower airways presents during the course of an exacerbation and treatment.

**Methods:**

We recruited children (aged 1-15) undergoing antibiotic treatment for bronchiectasis exacerbations at Starship Children’s Hospital and outpatient clinics. Sputum and nasal swabs were taken before and after antibiotic treatment. Sample DNA was extracted, then bacterial 16S rRNA genes amplified and sequenced *via* Illumina MiSeq.

**Results:**

Thirty patients were recruited into this study with 81 samples contributing to the final dataset, including 8 patients with complete sets of upper and lower airway samples at both (before and after antibiotics) timepoints. Changes in alpha-diversity over the course of an exacerbation and treatment were non-significant. However, sample composition did alter over the course of an exacerbation, with most notably a reduction in the relative abundance of amplicon sequence variants assigned to *Haemophilus*.

**Discussion:**

*Haemophilus* has been associated with more severe symptoms in respiratory infections and a reduction in its relative abundance may represent a positive shift in a patient’s microbiota. Current treatments for bronchiectasis may preserve bacterial diversity while altering microbiota composition.

## Introduction

Non-cystic fibrosis bronchiectasis (hereafter bronchiectasis) is increasingly reported in children across the world and represents a significant public health problem in New Zealand. Bronchiectasis had a reported incidence of 13.2 per 100,000 New Zealanders in 2017, some 3.5 times greater than that reported in 2002, with hospitalisations increasing by 49% in a similar timeframe (Twiss et al., 2005; Barnard and Zhang, 2018). This disease is particularly common within disadvantaged communities and associated with environmental factors such as overcrowding (Singleton et al., 2014). Bronchiectasis is traditionally described as a chronic disease involving permanent dilatation of the respiratory tract (Chang et al., 2018), however in children dilatation appears to be somewhat reversible, at least for some specific aetiologies (Gaillard et al., 2003). To understand what causes bronchiectasis to either resolve or persist, and to take advantage of the potential therapeutic window available in childhood, investigating changes in the respiratory health of affected individuals over time is critical.

Exacerbations are a key characteristic of bronchiectasis disease progression, whereby a patient experiences symptoms such as worsening cough, breathlessness or increased sputum volume (Hill et al., 2017). Exacerbations have been used as a marker of disease severity, with more frequent and longer-lasting exacerbations correlating with poorer prognoses (Chang et al., 2018). Additionally, exacerbations drive a vicious cycle of disease progression whereby each exacerbation makes future episodes both more likely and more severe (Cole., 1986) although it is unclear what causes exacerbations, microbes may play a role *via* proliferation of pathogens increasing airway inflammation (Amati et al., 2019).

Prolonged courses of high-dose antibiotics are frequently prescribed for exacerbations, and while sputum microbiological culture may guide clinical decisions, antibiotic sensitivity results do not necessarily predict clinical response (Chang et al., 2010). Studying the respiratory microbiota (i.e. the complex microbial community which exists throughout the respiratory tract) may help explain this lack of concordance. Particular microbial communities have been associated with bronchiectasis severity in adults and used to predict likelihood of future exacerbations (Rogers et al., 2014b). In comparable airway diseases such as cystic fibrosis and chronic obstructive pulmonary disease, exacerbations and their treatment have been reported to alter the microbiota (Carmody et al., 2013; Tiew et al., 2021). Particularly notable is the potential for antibiotic treatment to increase the relative abundance of *Pseudomonas aeruginosa* in individuals whose microbial communities were not initially dominated by this microbe (Rogers et. al., 2014a). It should be noted, however, that both the degree and resilience of these changes, particularly after treatment is stopped, remain a source of debate (Bevivino et al., 2019).

Longitudinal studies of adult bronchiectasis show long-term stability in the microbiota (Woo et al., 2019), with limited, potentially transient changes in the microbiota observed with exacerbation and treatment (Tunney et al., 2013). These changes commonly involve reductions in the relative abundance of dominant, core taxa (Tunney et al., 2013), with compositional changes primarily limited to less well-adapted, satellite taxa (Purcell et al., 2014). Despite the seeming stability of the bronchiectasis microbiota in adulthood, these studies reaffirm the capacity of the microbiota for predicting future disease severity given the potential malleability of the microbiota in childhood (Cox et al., 2019; Pillarisetti et al., 2019). It is therefore imperative to determine whether compositional changes may be introduced during exacerbations and treatments in children and what effect this might have on subsequent clinical outcomes.

In a previous study we demonstrated that the microbiotas of children with bronchiectasis at the point of clinical diagnosis were similar to those of healthy individuals without respiratory disease (Pillarisetti et al., 2019). While providing a clear baseline measurement, an important consideration was that the children were clinically well at the time. In this study we compared the lower and upper airway microbiotas of paediatric patients with bronchiectasis undergoing an exacerbation, both before and after treatment.

## Methods

We undertook a prospective observational study, recruiting children with non-CF bronchiectasis undergoing antibiotic treatment for exacerbation admitted to Starship Children’s Hospital Ward (Auckland, New Zealand) or one of the outpatient clinics between June 2015 and July 2019. Patients were included if they were between the ages of 1-15 years, known to have bronchiectasis on high resolution computed tomography, and were judged to have the clinical symptoms of a bronchiectasis exacerbation by clinical staff resulting in either inpatient treatment or regular reviews at the Starship Bronchiectasis Clinic. Patients were excluded if they had a diagnosis of cystic fibrosis, history of immunodeficiency, significant developmental delay, chromosomal abnormality or had previously received bone marrow treatment. Upper (nasal swab) and lower airway (sputum) samples were collected by clinical staff. Samples were collected before and after antibiotic treatment, with the first sample being taken before the first antibiotic treatment and the last sample being taken on the day this treatment was stopped. Ethics approval was granted by the Northern B Health and Disability Ethics Committee (ref: 15/NTB/7).

Laboratory analysis was performed in line with our previous study (Pillarisetti et al., 2019). Briefly, swab samples were stored in RNA*later* while sputum was centrifuged to produce a pellet for nucleic acid extraction. This initial processing was performed on the day of sample collection by LabPlus, the Auckland City Hospital laboratory. Samples were then stored at -80°C prior to further processing. DNA extraction was performed using pre-prepared bead-beating tubes containing ~0.5 g of 0.1 mm diameter silica beads and four 1 mm diameter glass beads in conjunction with the Qiagen AllPrep DNA/RNA Isolation Kit. 16S rRNA gene amplicons (V3-V4 region) were generated using the primer pair 341F/785R and visualized using gel electrophoresis. Samples for which PCR products could be observed were then purified using AMPure beads and sequenced by Auckland Genomics Ltd using Illumina MiSeq. Negative extraction controls, comprising sterile water, were processed alongside samples and did not produce a substantial number of sequence reads (0-426). Sequence data are available *via* Genbank (PRJNA683148). Amplicon sequence variants (ASVs) were generated by DADA2 in R, quality filtered using the filterandTrim command (minLen=15, maxEE (3,3), truncQ=2) and assigned *via* the SILVA (v138) taxonomic database. Prior to rarefaction all ASVs which were not assigned to a bacterial lineage (Eukaryota, Archaea and Unknown) were removed. Following rarefaction to 2616 reads/sample, alpha- (vegan::diversity, vegan::estimateR) and beta-diversity (vegan::vegdist) were calculated using the vegan package in R. For differences in alpha-diversity datasets we first evaluated for normality using the Shapiro-Wilks test (stats::shapiro.test), with a paired t-test (stats:: t.test) for the Shannon diversity metric and Wilcoxon rank-sum test (stats::wilcox.test) used for the observed number of ASVs metric. The influence of metadata characteristics on Bray-Curtis beta-diversity was calculated by using PERMANOVA (vegan::adonis) with the patient identifier first fitted to the model to account for inter-individual variability. Differentially abundant taxa were identified both *via* linear discriminant analysis effect size (LEfSe) and DESeq2. The LEfSe analysis was performed with a threshold on the logarithmic LDA score of 2.0 and the alpha values for the Kruskal-Wallis factorial test and pairwise Wilcoxon test set to 0.05; for both LEfSe and DESeq2 differential abundance was considered significant if the p-value was below 0.05.

## Results

In total, 99 samples were collected from 30 patients (**Table 1**). The reason that 120 samples (i.e. 30 patients x 2 timepoints x 2 sample types) could not be collected was due to both a loss of patients to follow-up or early discharge as well as refusal to provide a cough suction sample. The recruited cohort had a median age of 5.6 years (range: 1-15 years) with Māori (37%) and Pasifika (60%) children being over-represented in the patient population, likely reflecting the increased disease burden in these populations. All included patients were given some form of antibiotic treatment, with 36% receiving oral antibiotics and 64% intravenous antibiotics for a median of 14 days. 16S rRNA gene sequence data were successfully obtained from 81 samples: this included 8 patients with complete sets of 4 samples (before/after treatment, upper/lower airway), as well as 28 upper/lower airway pairs and 29 before/after treatment pairs. Seventeen of the 18 samples lost to laboratory processing failed to generate a PCR product and were not submitted for sequencing, while one failed sequence quality filtering. Overall, 3976737 sequence reads, corresponding to 3928 ASVs, were obtained with a range of 2616-137475 total reads per sample. Post-rarefying to 2616 reads/sample, a total of 2114 ASVs remained. ASVs which were identified in negative extraction controls are shown in **Table S1**. The most abundant and prevalent ASVs across the entire dataset were affiliated with common airway taxa (**Figures S1, S2**), including *Moraxella* (108 ASVs, 23% average relative abundance), *Streptococcus* (147 ASVs, 16%), *Haemophilus* (119 ASVs, 15%) and *Neisseria* (118 ASVs, 9%). Additionally, 37 ASVs were assigned to *Pseudomonas*, however these totalled less than 1% average relative abundance.

**Table 1:** Patient sample and demographics data.

Patient ID	Age (years)	Sex	Ethnicity	Nasal samples		Sputum samples		Duration between samples (days)	Treatment antibiotic	Prophylaxis antibiotic
				Pre- treatment	Post-treatment	Pre-treatment	Post-treatment			
**EXB1**	4.1	F	Pasifika	Y	Y	Y	NC	14	Oral Augmentin TDS	
**EXB2**	7.8	M	Pasifika	Y	NC	Y	NC	NA	IV Augmentin Q8H	
**EXB3**	5.5	M	Pasifika	UN	Y	Y	UN	14	Oral Augmentin TDS	
**EXB4**	3	M	Pasifika	Y	Y	Y	Y	12	Oral Augmentin TDS	
**EXB5**	2.6	M	Māori	Y	Y	Y	UN	10	IV Augmentin Q8H	
**EXB6**	5.6	F	Māori	Y	Y	Y	Y	14	Oral Augmentin TDS	
**EXB7**	11.5	M	Māori	Y	Y	UN	Y	17	IV Cefuroxime Q8H	
**EXB8**	10.7	M	Pasifika	UN	Y	UN	Y	13	Oral Amoxycillin TDS	
**EXB9**	5.5	F	Māori	Y	Y	Y	Y	10	IV Cefuroxime Q8H	*Oral Erythromycin BD
**EXB10**	10.6	M	Māori	NC	Y	UN	Y	9	IV Augmentin Q8H	Oral Roxithromycin OD
**EXB11**	12.6	F	Pasifika	Y	Y	UN	UN	11	IV Augmentin Q8H	
**EXB12**	7.1	F	Pasifika	Y	Y	UN	UN	12	IV Cefuroxime Q8H	*Oral Augmentin BD
**EXB13**	3	M	Pasifika	Y	Y	Y	Y	6	Oral Azithromycin OD	
**EXB14**	4.7	M	Pasifika	Y	NC	Y	Y	9	IV Augmentin Q8H	
**EXB15**	11.5	M	Māori	Y	UN	UN	Y	8	IV Augmentin Q8H	
**EXB16**	6.6	M	Pasifika	Y	Y	UN	UN	18	Oral Augmentin TDS	
**EXB17**	1.5	M	Māori	NC	Y	Y	Y	20	IV Cefuroxime Q8H	
**EXB18**	2.3	M	Māori	NC	NC	Y	Y	10	IV Augmentin Q8H	
**EXB19**	1.9	F	Pasifika	Y	NC	Y	NC	NA	Oral Augmentin TDS	
**EXB20**	4	M	Māori	NC	NC	Y	Y	6	IV Augmentin Q8H	
**EXB21**	2.3	M	Pasifika	Y	Y	Y	Y	14	Oral Augmentin TDS	
**EXB22**	2.1	F	Pasifika	Y	Y	Y	Y	14	Oral Augmentin TDS	
**EXB23**	4.8	F	Māori	Y	Y	NC	Y	13	IV Augmentin Q8H	
**EXB24**	14	F	Asian	Y	Y	Y	Y	10	IV Cefuroxime Q8H	
**EXB25**	3.6	M	Pasifika	Y	Y	Y	Y	29	Oral Augmentin TDS	
**EXB26**	11.9	M	Pasifika	NC	NC	Y	NC	NA	IV Augmentin Q8H	Oral Azithromycin OD
**EXB27**	13.5	M	Pasifika	NC	NC	Y	Y	9	IV Augmentin Q8H	
**EXB28**	11.4	M	Pasifika	NC	NC	Y	NC	NA	IV Cefuroxime Q8H	
**EXB29**	11	F	Māori	Y	UN	Y	UN	13	IV Cefuroxime Q8H	
**EXB30**	15	F	European	Y	UN	Y	Y	14	IV Cefuroxime Q8H	

Key demographics of patient population. *Indicates that prophylaxis antibiotic treatment was suspended while this patient was on the exacerbation treatment. Y, Sequence data available for analysis; NC, Sample not collected; UN, Sample was collected but failed to generate usable sequence data. IV, intravenous; NA, not available.

As we did not have complete sample pairs (i.e. before vs after treatment for a given sample site; nasal vs sputum for a given timepoint) for every patient (as shown in **Table 1**), for all subsequent analyses we used only subsets of data comprising complete pairs to ensure validity of our statistical analyses. Thus, for analyses of treatment/exacerbation effects we included n=15 pairs for nasal samples and n=14 sample pairs for sputum. For explicit comparisons of nasal vs sputum microbiotas, we included n=15 sample pairs for timepoint 1 (pre-treatment) and n=13 pairs for timepoint 2 (post-treatment).

### Effects of Treatment and Exacerbation on the Nasal and Sputum Microbiotas

**Figure 1:**
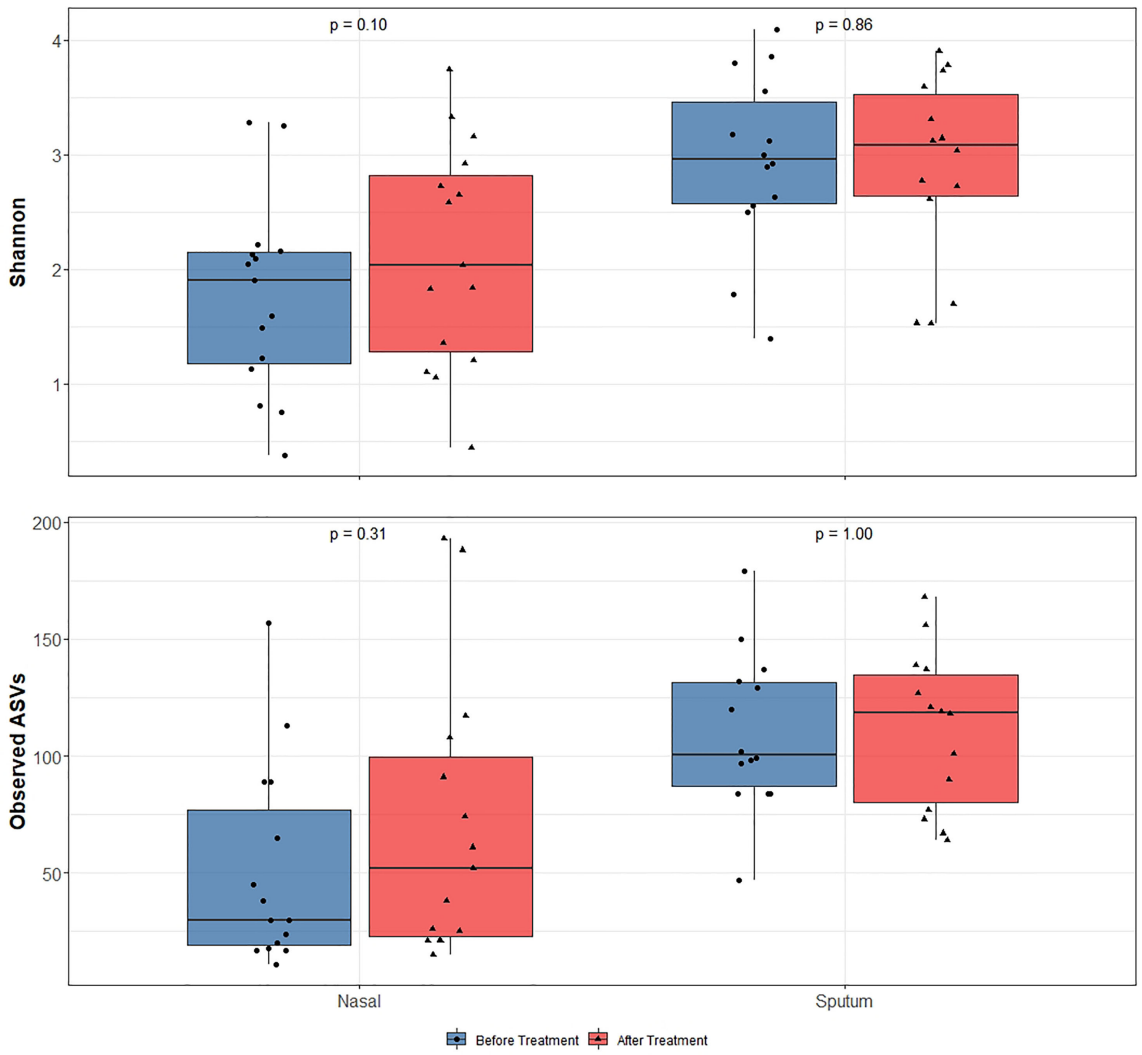
16S rRNA gene-based bacterial alpha-diversity in paired before and after treatment for bronchiectasis exacerbation in paediatric patients. After assessing data for normality using the Shapiro-Wilks test, significance was calculated by using a paired t-test for the Shannon diversity metric and Wilcoxon rank-sum test for the observed number of ASVs. Diversity was higher in sputum samples than nasal samples but diversity did not significantly change following antibiotic treatment in any sample type.

**Figure 2:**
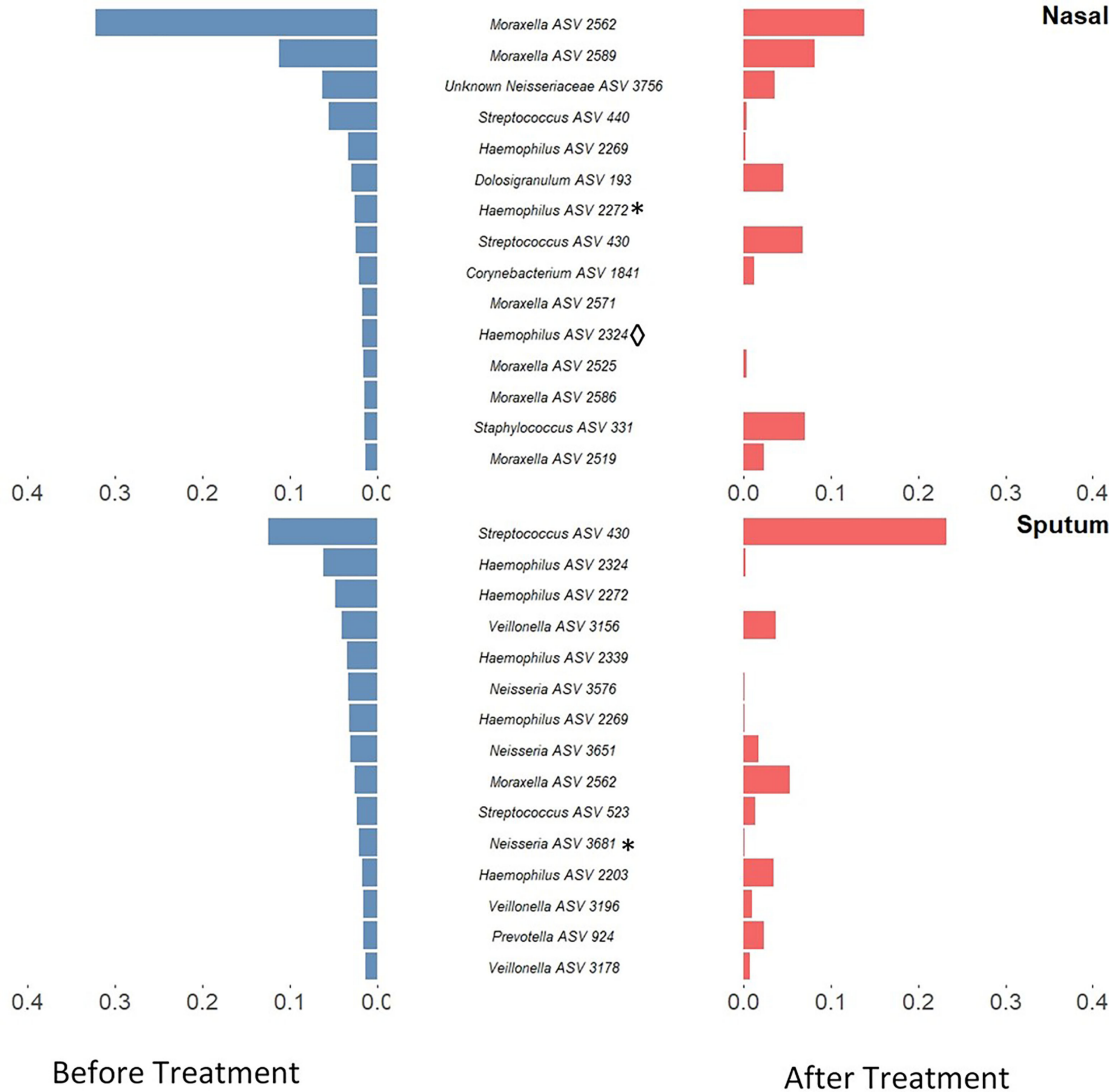
Rank-abundance plots showing the 15 most abundant 16S rRNA gene ASVs in paired samples from before treatment (blue) and their respective abundance after treatment (red). ASVs are ranked based on their relative sequence abundance before treatment. Taxonomic composition was altered in both sputum and nasal samples with a notable decrease of *Haemophilus* ASVs in sputum samples and *Moraxella* in nasal samples. Those ASVs which were identified as significantly differentially abundant by DESeq2 are marked by * while those which were identified as significantly differentially abundant by LEfSE are denoted by ◊. An inverse version of this plot, whereby ASVs are ranked based on their relative sequence abundance *after* treatment, can be found in **Figure S3**.

**Figure 3:**
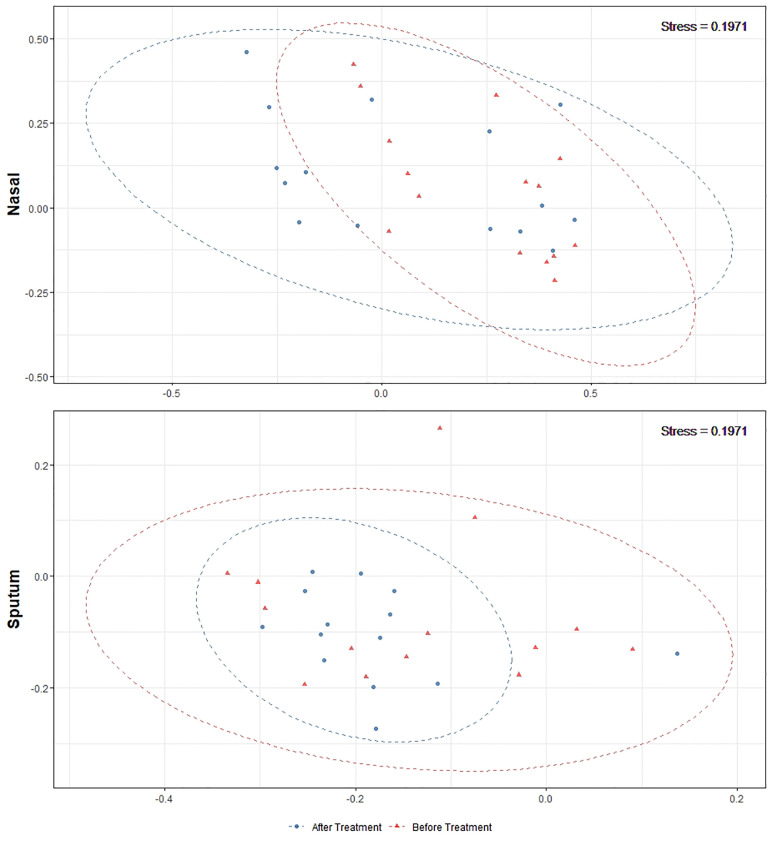
Non-metric multidimensional scaling plots of 16S rRNA gene-based beta-diversity using the Bray-Curtis metric for bacteria in paired nasal and sputum samples across timepoints. Bacterial community dispersion was largely unchanged in nasal samples following treatment but was reduced in sputum samples. The confidence ellipses capture 95% of the relevant data points.

Alpha-diversity of the sputum and anterior nares microbiotas showed a slight, but non-significant, tendency to increase following treatment (**Figure 1**). This applied to both the Shannon and observed ASVs diversity metrics. We next sought to determine whether any of the most dominant ASVs changed in relative abundance during the course of the exacerbation and treatment. Among the 15 most abundant ASVs at the first timepoint, we saw non-significant decreases in the dominant ASVs assigned to *Moraxella* in nasal samples (i.e. ASVs 2562, 2589), and a non-significant increase post-treatment in the dominant *Streptococcus* ASV (ASV 430) in sputum (**Figure 2**). The only ASVs that did differ significantly among those shown in **Figure 2** were *Haemophilus* ASVs 2324 (which decreased in nasal samples according to LEfSe but not DESeq2) and 2272 (which decreased in nasal samples in DESeq 2 only), as well as *Neisseria* ASV 3681 which was identified by DESeq2 analysis as decreasing in the sputum samples. Several other, less abundant ASVs (not shown in **Figure 2**) – including further *Haemophilus* ASVs – also differed significantly according to LEfSe and/or DESeq2 (**Tables S2**–**S5**). To capture any ASVs that were dominant post-treatment but were not among the 15 most abundant ASVs pre-treatment, we assembled another rank-abundance plot to show these (**Figure S3**). Beta-diversity, encapsulating the differences between bacterial communities, was assessed using Bray-Curtis dissimilarity. Bacterial communities were largely overlapping between pre- and post-treatment timepoints, for both nasal and sputum samples (**Figure 3**). Neither anatomical site nor treatment had a statistically significant influence on beta-diversity as assessed by PERMANOVA.

### Comparing the Nasal and Sputum Microbiotas

Alpha-diversity was significantly higher in sputum compared with nasal samples, using paired samples at each time point (**Figure S4**). As outlined in **Tables S6–S9**, many ASVs were identified as differentially abundant between nasal and sputum samples *via* LEfSe and DESeq2 analyses. Among notable examples, *Corynebacterium*- and *Dolosigranulum*-affiliated ASVs tended to be characteristic of nasal samples in differential abundance analyses, whereas *Streptococcus* and *Haemophilus* ASVs were among those identified as differentially abundant in sputum. In ordination plots, separation of bacterial communities was clearer across anatomical sites than across the different timepoints (**Figures S5, S6**).

## Discussion

Whilst controversial, higher bacterial diversity is often interpreted as reflective of improved human health (Man et al., 2017). With the use of antibiotics for respiratory exacerbations in this patient cohort we noticed a slight (albeit non-significant) increase in bacterial diversity with treatment. While any increase in diversity was marginal, the notable finding here is that diversity did not *decrease* despite antibiotic treatment. This may in part be reflective of a ‘bloom’ whereby a particular pathogen overgrows and comes to dominate the bacterial community, then less abundant community members bounce back once said pathogen is removed or at least reduced by antibiotic treatment. Given the oft-stated association of bacterial diversity with health (Man et. al., 2017), and antibiotic treatment in these patients being ceased only on improvement of exacerbation symptoms judged by the attending physician, this may reflect an improvement in the overall respiratory health of these patients.

Antibiotic treatments may also create a selection pressure to drive microbial communities towards a common type, as seen by the tighter clustering (on the ordination plot) of post-treatment sputum samples. This change, together with the compositional changes observed in these microbiotas, reflects the potential malleability of the respiratory microbiota in childhood. The most notable change was a reduction in both number and proportional abundance of multiple ASVs corresponding to *Haemophilus*, a taxon commonly associated with the acute infections which often underlie exacerbations (Amati et al., 2019); *Haemophilus* ASVs were also identified as differentially abundant in LEfSe and/or DESeq2 analyses. These changes paired with the stability in diversity could be an indicator of overall improving respiratory health in these patients.

While the findings reported here are promising, several limitations do warrant caution in further interpreting these results. In addition to low sample numbers, treatment and assessment of symptoms was non-standardised as it was based on clinician judgement. Additionally, the use of 16S rRNA amplicon data limit the taxonomic resolution which could be assessed despite the fact that species- or strain-level resolution could lead to valuable insights. Direct quantification through the use of real-time PCR or droplet digital PCR could also be useful for both determining changes in overall bacterial load and taxa of interest such as *Haemophilus*. Finally, this study was only conducted over a short period of time and follow-up studies will be required in order to determine both the long-term resilience of the microbiota following an exacerbation and to identify those changes which precede an exacerbation.

This small-scale pilot study offers some promising results, particularly regarding the lack of decrease, and even marginal increase, in bacterial diversity following treatment for bronchiectasis exacerbations. The reduction of *Haemophilus* ASVs is suggestive of a positive therapeutic effect, given the association of this genus with infection and severity in other studies, making this an exciting avenue for future research. Pairing these findings with the greater malleability of respiratory microbiotas in paediatric patients highlights the importance of early intervention with a potential to reverse the disease.

## Data Availability Statement

The datasets presented in this study can be found in online repositories. The names of the repository/repositories and accession number(s) can be found below: https://www.ncbi.nlm.nih.gov/, PRJNA683148.

## Ethics Statement

The studies involving human participants were reviewed and approved by Northern B Health and Disability Ethics Committee. Written informed consent to participate in this study was provided by the participants’ legal guardian/next of kin.

## Author Contributions

DB: laboratory processing of samples, bioinformatic analysis of sequence data, and writing of manuscript. TR: laboratory processing of samples and analysis design. AA: patient recruitment and sampling, gathering clinical patient data, and feedback on manuscript. MT: experimental design and feedback on manuscript. NP: experimental design, patient recruitment and sampling, gathering clinical patient data, and feedback on manuscript.

## Funding

We gratefully acknowledge funding from the Asser Trust, A+ Trust, Athlae Lyon Starship Research Trust and Starship foundation. We also gratefully acknowledge the children, families and bronchiectasis team and Starship Hospital for their participation and facilitation of the study.

## Conflict of Interest

The authors declare that the research was conducted in the absence of any commercial or financial relationships that could be construed as a potential conflict of interest.

## Publisher’s Note

All claims expressed in this article are solely those of the authors and do not necessarily represent those of their affiliated organizations, or those of the publisher, the editors and the reviewers. Any product that may be evaluated in this article, or claim that may be made by its manufacturer, is not guaranteed or endorsed by the publisher.
