# Supplementary material for: Dynamic Upper and Lower Airway Microbiotas in Paediatric Bronchiectasis Exacerbations: A Pilot Study
Source: Front Cell Infect Microbiol. 2022 Jan 24;11:773496. doi: 10.3389/fcimb.2021.773496 (PMC8818954; doi:10.3389/fcimb.2021.773496)
Supplement: Supplementary file 1 [file Table_1.docx]

**Dynamic upper and lower airway microbiotas in paediatric bronchiectasis exacerbations: a pilot study**

**Online Supplement**

**Figure S1:** Taxa plot of nasal samples showing the 15 ASVs with the highest average relative abundance across all samples

**Figure S2:** Taxa plot of sputum samples showing the 15 ASVs with the highest average relative abundance across all samples

Before Treatment

After Treatment


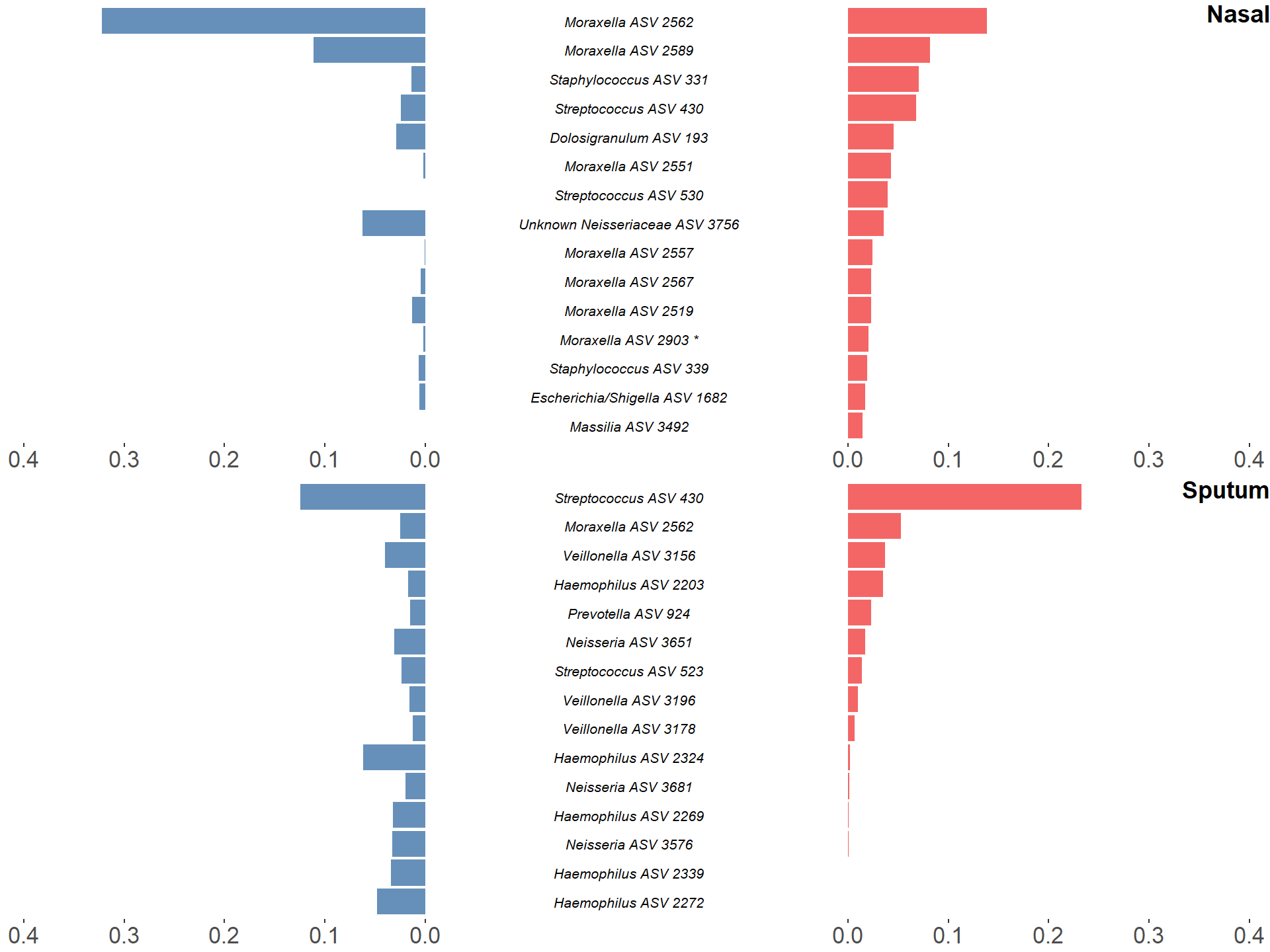


*

*

*

**Figure S3:** Rank-abundance plots showing the 15 most abundant 16S rRNA gene amplicon sequence variants in paired samples from after treatment (red) and their respective abundance before treatment (blue). ASVs are ranked based on their relative sequence abundance after treatment. Taxonomic composition was altered in both sputum and nasal samples with a notable decrease of *Haemophilus* ASVs in sputum samples and *Moraxella* in nasal samples. Those ASVs which were identified as significantly differentially abundant by DESeq2 were marked by *. None of these particular ASVs were identified as differentially abundant by LEfSe.

**Figure S4:** 16S rRNA gene-based bacterial alpha-diversity in paired sputum and nasal samples both before and after treatment for bronchiectasis exacerbation in paediatric patients.
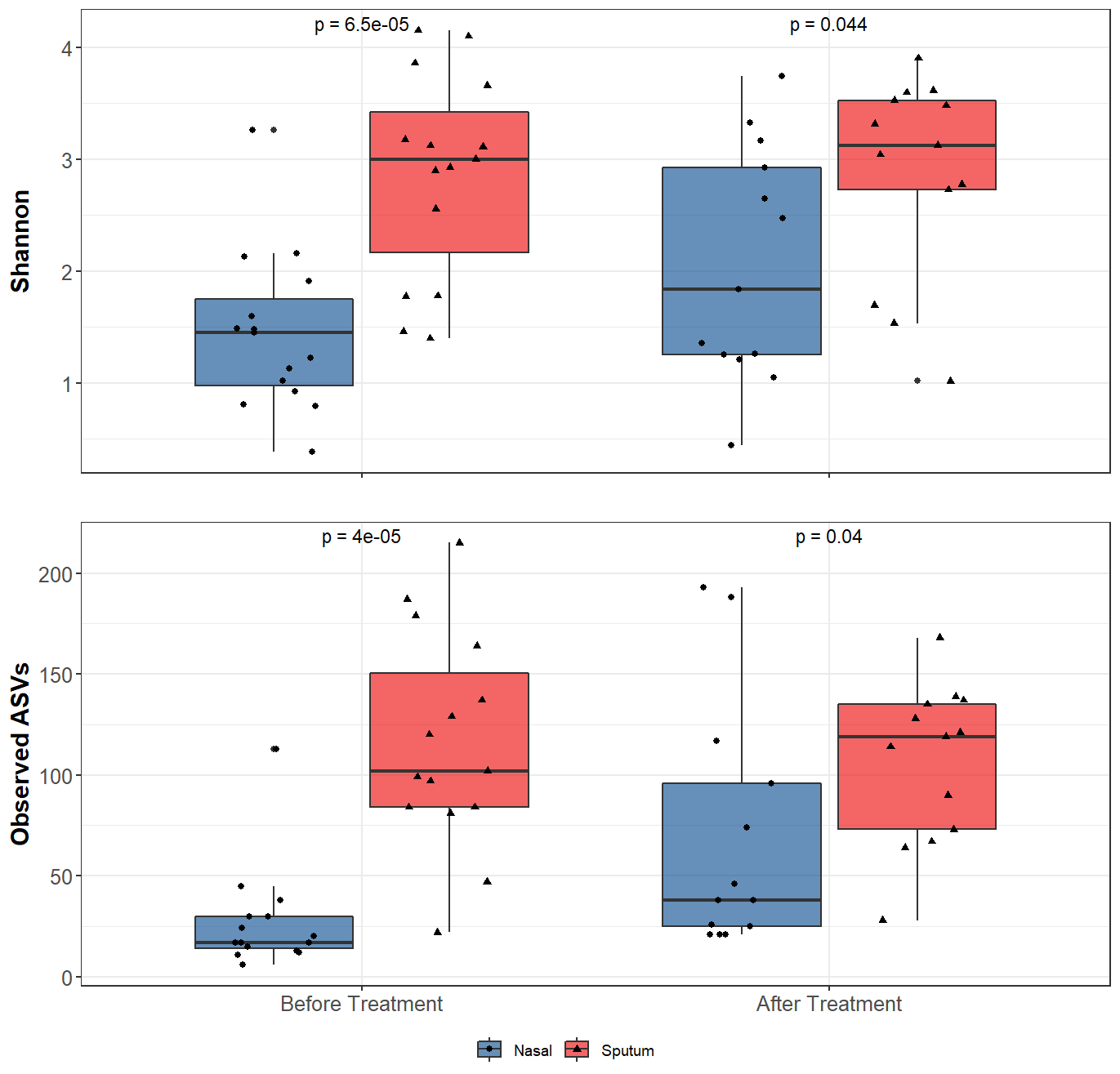
After assessing data for normality using the Shapiro-Wilks test, significance was calculated by using a paired t-test for the Shannon diversity metric and Wilcoxon rank-sum test for the observed number of ASVs.

**Figure S5:** Non-metric multidimensional scaling plots of 16S rRNA gene-based beta-diversity using the Bray-Curtis metric for bacteria in nasal and sputum samples. The data shows bacterial community dispersion across sample site. The ellipses represent a confidence ellipse capturing 95% of the relevant data points.


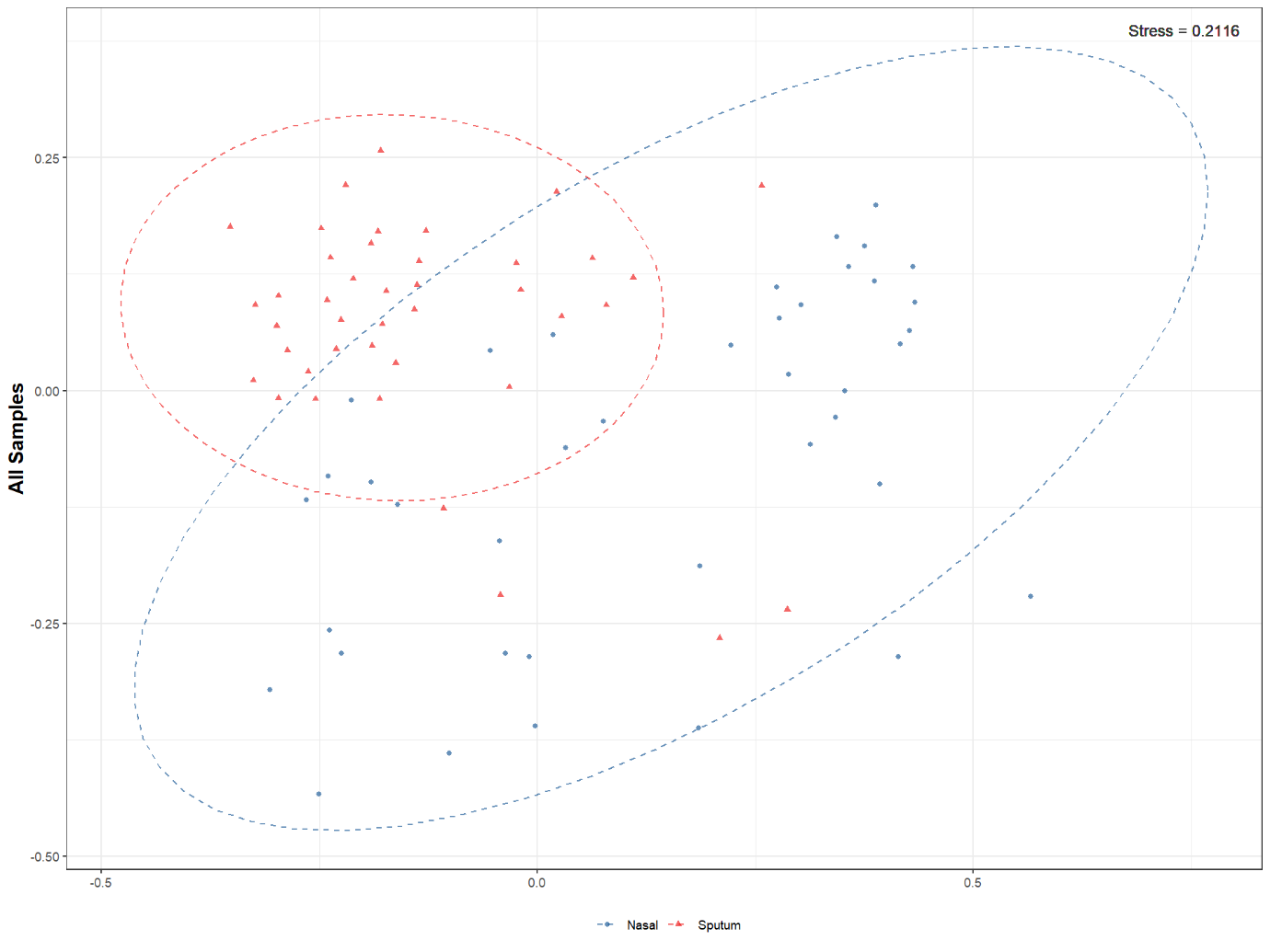

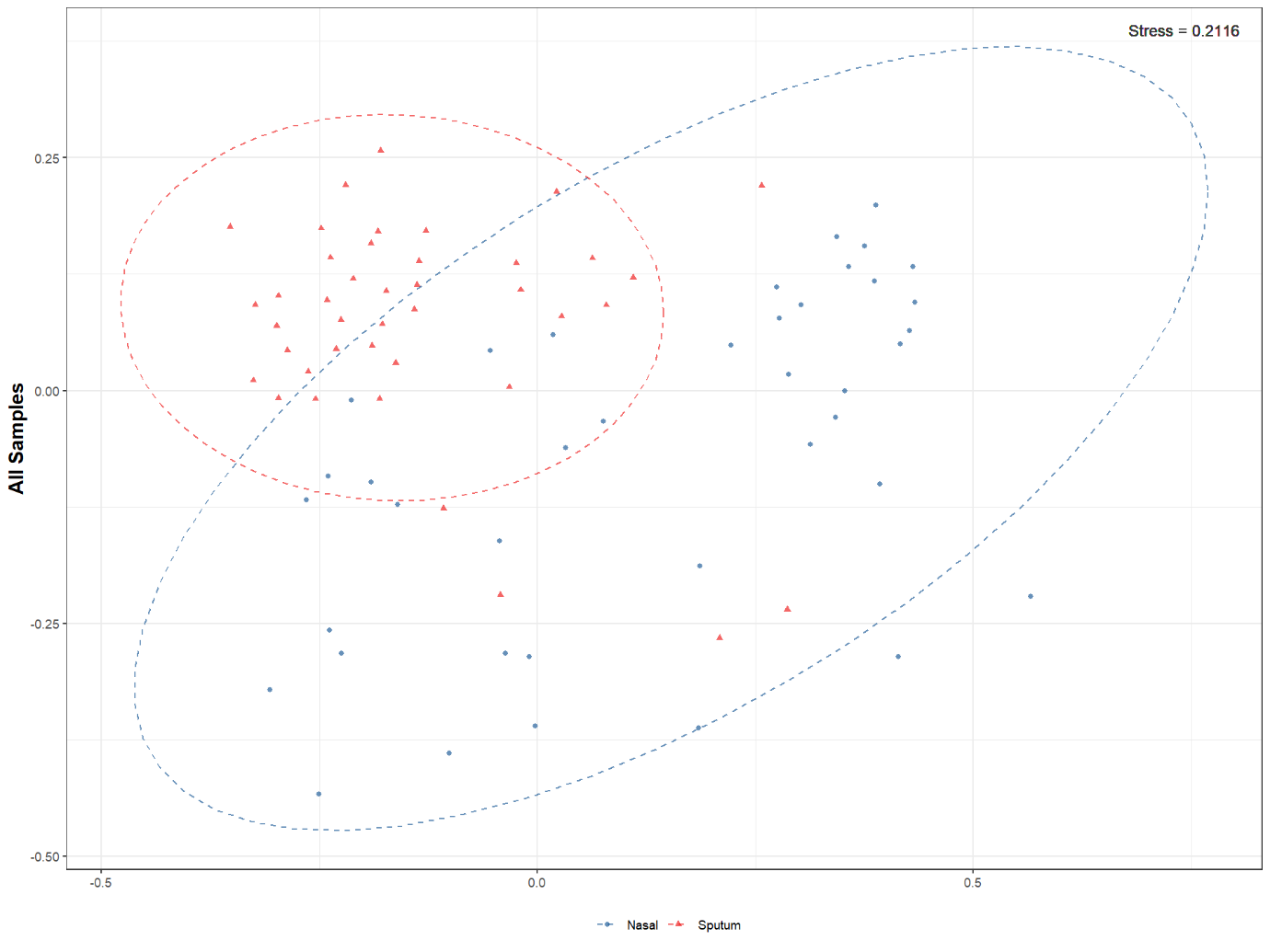

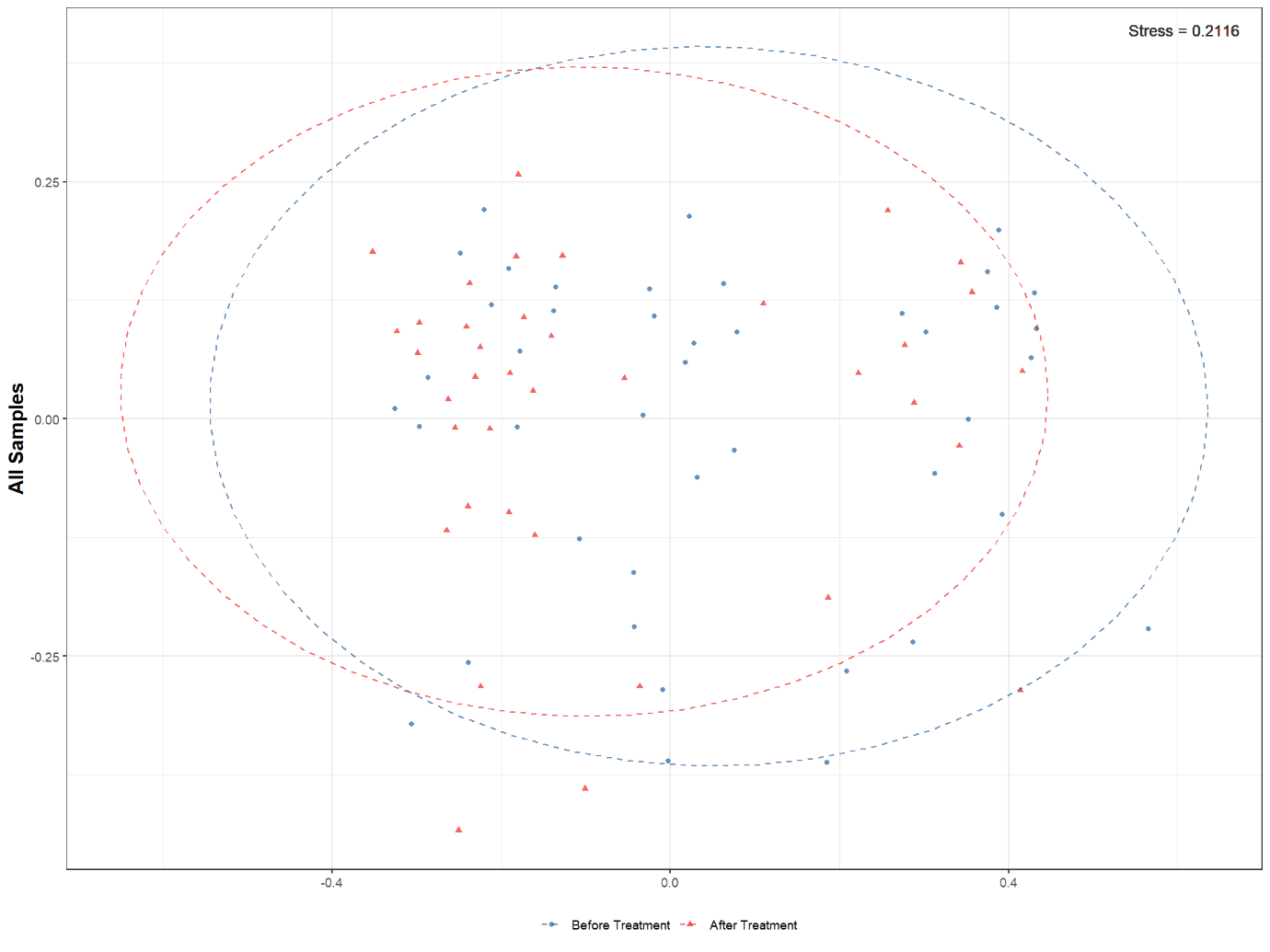

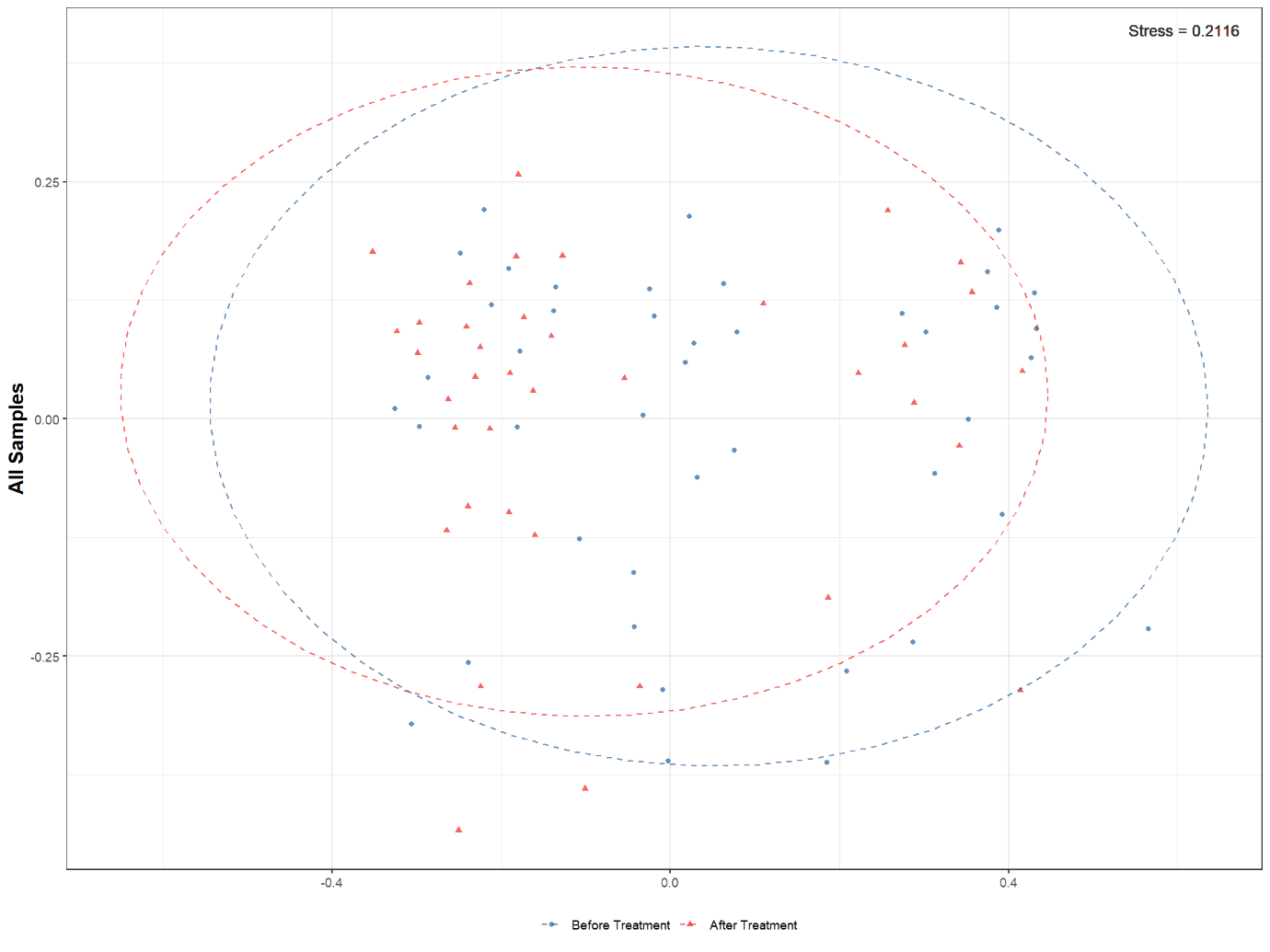


**Figure S6:** Non-metric multidimensional scaling plots of 16S rRNA gene-based beta-diversity using the Bray-Curtis metric for bacteria in nasal and sputum samples. There is no clear bacterial community separation across timepoint. The ellipses represent a confidence ellipse capturing 95% of the relevant data points.

| Genus | Blank 1 | Blank 2 | Blank 3 | Blank 4 | Blank 5 | Blank 6 | Blank 7 | Blank 8 | Blank 9 | Blank 10 |
| --- | --- | --- | --- | --- | --- | --- | --- | --- | --- | --- |
| Unknown *Alphaproteobacteria* | 0 | 2 | 0 | 0 | 0 | 0 | 0 | 0 | 0 | 0 |
| *Lactobacillus* | 0 | 0 | 0 | 0 | 4 | 0 | 0 | 0 | 0 | 0 |
| *Streptococcus* | 0 | 0 | 0 | 0 | 0 | 0 | 37 | 0 | 0 | 0 |
| *Escherichia/Shigella* | 0 | 0 | 0 | 0 | 0 | 0 | 308 | 8 | 5 | 0 |
| *Escherichia/Shigella* | 0 | 0 | 0 | 0 | 0 | 0 | 5 | 0 | 0 | 0 |
| *Escherichia/Shigella* | 7 | 0 | 0 | 0 | 0 | 0 | 0 | 0 | 0 | 0 |
| *Escherichia/Shigella* | 0 | 0 | 0 | 0 | 0 | 0 | 7 | 0 | 0 | 0 |
| *Robinsoniella* | 0 | 0 | 0 | 0 | 0 | 5 | 0 | 0 | 0 | 0 |
| *Tyzzerella* | 0 | 0 | 0 | 0 | 0 | 0 | 69 | 0 | 0 | 0 |
| *Lachnoanaerobaculum* | 8 | 0 | 0 | 0 | 0 | 0 | 0 | 0 | 0 | 0 |

**Table S1: Raw count table of sequences derived from extraction blanks processed alongside samples.**

**Table S2 LefSE output for paired nasal samples across time**

| Taxa | Marker | p value |
| --- | --- | --- |
| Haemophilus_ASV_2324 | Before Treatment | 0.003832 |
| Haemophilus_ASV_2321 | Before Treatment | 0.017717 |
| Rothia_ASV_1731 | After Treatment | 0.035248 |
| Veillonella_ASV_3148 | After Treatment | 0.03519 |
| Lactococcus_ASV_359 | After Treatment | 0.016594 |
| Micrococcus_ASV_1590 | After Treatment | 0.016537 |
| Veillonella_ASV_3144 | After Treatment | 0.040995 |
| Subdoligranulum_ASV_3871 | After Treatment | 0.041077 |
| Blautia_ASV_1954 | After Treatment | 0.046991 |
| Staphylococcus_ASV_310 | After Treatment | 0.035248 |

**Table S3 LefSE output for paired sputum samples across time**

| Taxa | Marker | p value |
| --- | --- | --- |
| Saccharimonadaceae_TM7x_ASV_1435 | Before Treatment | 0.034494 |
| Haemophilus_ASV_2304 | Before Treatment | 0.029615 |
| Fusobacterium_ASV_2396 | Before Treatment | 0.015984 |
| Fusobacterium_ASV_2418 | Before Treatment | 0.046159 |
| Leptotrichia_ASV_2446 | Before Treatment | 0.021673 |
| Capnocytophaga_ASV_756 | After Treatment | 0.047133 |
| Actinomyces_ASV_1783 | After Treatment | 0.015887 |
| Alloprevotella_ASV_1125 | After Treatment | 0.034494 |

**Table S4: DESEq2 output for paired nasal samples across time**

| TaxaName | baseMean | log2FoldChange | lfcSE | stat | pvalue | padj |
| --- | --- | --- | --- | --- | --- | --- |
| Staphylococcus_ASV_339 | 10.43603 | 4.246397 | 0.844312 | 5.029418 | 4.92E-07 | 6.84E-05 |
| Haemophilus_ASV_2272 | 5.58357 | 2.729272 | 0.757602 | 3.602512 | 0.000315 | 0.021903 |
| Moraxella_ASV_2903 | 3.80317 | -2.54004 | 0.761108 | -3.33729 | 0.000846 | 0.039197 |

**Table S5: DESEq2 output for paired sputum samples across time**

| TaxaName | baseMean | log2FoldChange | lfcSE | stat | pvalue | padj |
| --- | --- | --- | --- | --- | --- | --- |
| Haemophilus_ASV_2177 | 5.347449 | -2.51644 | 0.709413 | -3.54722 | 0.000389 | 0.02851 |
| Streptobacillus_ASV_2467 | 3.734868 | 2.48089 | 0.713105 | 3.478998 | 0.000503 | 0.02851 |
| Neisseria_ASV_3681 | 4.803994 | 2.795374 | 0.80449 | 3.474716 | 0.000511 | 0.02851 |
| Neisseria_ASV_3809 | 5.031558 | 3.087448 | 0.808026 | 3.820978 | 0.000133 | 0.02851 |
| Neisseria_ASV_3565 | 5.069385 | 2.597593 | 0.79194 | 3.280036 | 0.001038 | 0.046292 |

**Table S6 LefSE output for paired samples before treatment**

| Taxa | Marker | p value |
| --- | --- | --- |
| Moraxella_ASV_2562 | Nasal | 0.025811 |
| Dolosigranulum_ASV_193 | Nasal | 7.47E-05 |
| Suttonella_ASV_2766 | Nasal | 0.016594 |
| Corynebacterium_ASV_1841 | Nasal | 0.000554 |
| Streptococcus_ASV_431 | Sputum | 0.02706 |
| Porphyromonas_ASV_1185 | Sputum | 0.007535 |
| Fusobacterium_ASV_2425 | Sputum | 0.035248 |
| Aggregatibacter_ASV_2225 | Sputum | 0.035248 |
| Streptococcus_ASV_443 | Sputum | 0.018454 |
| Veillonella_ASV_3148 | Sputum | 0.003188 |
| Porphyromonas_ASV_1190 | Sputum | 0.003301 |
| Neisseria_ASV_3593 | Sputum | 0.008797 |
| Granulicatella_ASV_278 | Sputum | 0.002009 |
| Prevotella_ASV_914 | Sputum | 0.03519 |
| Neisseria_ASV_3606 | Sputum | 7.45E-05 |
| Oribacterium_ASV_2023 | Sputum | 0.003282 |
| Granulicatella_ASV_263 | Sputum | 0.017633 |
| Neisseria_ASV_3651 | Sputum | 0.000179 |
| Streptococcus_ASV_416 | Sputum | 0.006133 |
| Haemophilus_ASV_2293 | Sputum | 0.016594 |
| Alloprevotella_ASV_809 | Sputum | 0.007562 |
| Streptococcus_ASV_511 | Sputum | 0.035248 |
| Prevotella_ASV_1097 | Sputum | 0.001378 |
| Porphyromonas_ASV_1177 | Sputum | 0.001389 |
| Bergeyella_ASV_690 | Sputum | 0.035131 |
| Veillonella_ASV_3170 | Sputum | 0.000551 |
| Bergeyella_ASV_705 | Sputum | 0.035767 |
| Fusobacterium_ASV_2381 | Sputum | 7.47E-05 |
| Prevotella_ASV_1274 | Sputum | 0.007548 |
| Streptococcus_ASV_436 | Sputum | 0.010906 |
| Streptobacillus_ASV_2479 | Sputum | 0.02518 |
| Lautropia_ASV_3442 | Sputum | 8.06E-05 |
| Streptococcus_ASV_552 | Sputum | 0.017443 |
| Haemophilus_ASV_2290 | Sputum | 0.009721 |
| Porphyromonas_ASV_1187 | Sputum | 0.001403 |
| Campylobacter_ASV_95 | Sputum | 0.000195 |
| Catonella_ASV_3855 | Sputum | 0.03519 |
| Absconditabacteriales__SR1__Unknown_Unknown_ASV_3320 | Sputum | 0.03519 |
| Veillonella_ASV_3099 | Sputum | 0.001553 |
| Streptococcus_ASV_548 | Sputum | 0.006041 |
| Prevotella_ASV_928 | Sputum | 0.035248 |
| Veillonella_ASV_3167 | Sputum | 0.03519 |
| Lachnospiraceae_Unknown_ASV_2734 | Sputum | 0.01648 |
| Megasphaera_ASV_3237 | Sputum | 0.035248 |
| Streptobacillus_ASV_2435 | Sputum | 0.035248 |
| Haemophilus_ASV_2220 | Sputum | 0.016594 |
| Neisseria_ASV_3611 | Sputum | 0.035248 |
| Haemophilus_ASV_2203 | Sputum | 2.18E-05 |
| Leptotrichia_ASV_2838 | Sputum | 0.03519 |
| Veillonella_ASV_3196 | Sputum | 0.000222 |
| Streptococcus_ASV_509 | Sputum | 0.015337 |
| Prevotella_ASV_1008 | Sputum | 0.007453 |
| Alloprevotella_ASV_1126 | Sputum | 0.000554 |
| Veillonella_ASV_3089 | Sputum | 0.03519 |
| Porphyromonas_ASV_1191 | Sputum | 0.02518 |
| Streptococcus_ASV_469 | Sputum | 0.02906 |
| Haemophilus_ASV_2304 | Sputum | 0.000554 |
| Streptococcus_ASV_523 | Sputum | 0.000793 |
| Streptobacillus_ASV_2467 | Sputum | 0.035248 |
| Veillonella_ASV_3178 | Sputum | 0.004884 |
| Prevotella_ASV_924 | Sputum | 0.000114 |
| Veillonella_ASV_3092 | Sputum | 0.003308 |
| Haemophilus_ASV_2178 | Sputum | 0.035248 |
| Haemophilus_ASV_2267 | Sputum | 0.007535 |
| Haemophilus_ASV_2191 | Sputum | 0.035248 |
| Streptococcus_ASV_415 | Sputum | 0.003265 |
| Porphyromonas_ASV_1180 | Sputum | 0.000376 |
| Haemophilus_ASV_2180 | Sputum | 0.015318 |
| Haemophilus_ASV_2175 | Sputum | 0.000209 |
| Haemophilus_ASV_2076 | Sputum | 0.016566 |
| Alloprevotella_ASV_956 | Sputum | 0.007548 |
| Neisseria_ASV_3600 | Sputum | 0.03519 |
| Haemophilus_ASV_2337 | Sputum | 0.000483 |
| Prevotella_ASV_1110 | Sputum | 0.004473 |
| Streptococcus_ASV_521 | Sputum | 0.001945 |
| Absconditabacteriales__SR1__Unknown_Unknown_ASV_3312 | Sputum | 0.016594 |
| Fusobacterium_ASV_2396 | Sputum | 0.003295 |
| Actinomyces_ASV_1619 | Sputum | 0.03519 |
| Neisseria_ASV_3627 | Sputum | 0.000209 |
| Neisseria_ASV_3613 | Sputum | 0.007562 |
| Streptobacillus_ASV_2459 | Sputum | 0.03519 |
| Campylobacter_ASV_88 | Sputum | 0.03519 |
| Abiotrophia_ASV_273 | Sputum | 0.011277 |
| Prevotella_ASV_1006 | Sputum | 0.007535 |
| Prevotella_ASV_1248 | Sputum | 0.016594 |
| Porphyromonas_ASV_1280 | Sputum | 0.035248 |
| Leptotrichia_ASV_2860 | Sputum | 0.003308 |
| Prevotella_ASV_1060 | Sputum | 0.016566 |
| Neisseria_ASV_3587 | Sputum | 0.007562 |
| Veillonella_ASV_3168 | Sputum | 0.003308 |
| Johnsonella_ASV_2733 | Sputum | 0.035248 |
| Fusobacterium_ASV_2418 | Sputum | 0.001383 |
| Streptococcus_ASV_450 | Sputum | 0.00269 |
| Porphyromonas_ASV_1282 | Sputum | 0.007562 |
| Prevotella_ASV_1063 | Sputum | 0.035248 |
| Rothia_ASV_1741 | Sputum | 0.035248 |
| Gemella_ASV_303 | Sputum | 0.002922 |
| Fusobacterium_ASV_2399 | Sputum | 0.007548 |
| Prevotella_ASV_1247 | Sputum | 0.03519 |
| Veillonella_ASV_3164 | Sputum | 0.003314 |
| Veillonella_ASV_3156 | Sputum | 0.000216 |
| Leptotrichia_ASV_2446 | Sputum | 0.007548 |
| Streptococcus_ASV_430 | Sputum | 0.000344 |
| Gemella_ASV_298 | Sputum | 0.026998 |
| Veillonella_ASV_3191 | Sputum | 0.035248 |
| Prevotella_ASV_1104 | Sputum | 0.016566 |
| Porphyromonas_ASV_1199 | Sputum | 0.007548 |
| Streptococcus_ASV_504 | Sputum | 0.001849 |

**Table S7 LefSE output for paired samples after treatment**

| Taxa | Marker | p value |
| --- | --- | --- |
| Corynebacterium_ASV_1858 | Nasal | 0.033775 |
| Staphylococcus_ASV_322 | Nasal | 0.033698 |
| Dolosigranulum_ASV_193 | Nasal | 0.002818 |
| Neisseriaceae_Unknown_ASV_3756 | Nasal | 0.033775 |
| Staphylococcus_ASV_331 | Nasal | 0.000411 |
| Cutibacterium_ASV_1886 | Nasal | 0.002818 |
| Helcococcus_ASV_3399 | Nasal | 0.033775 |
| Streptococcus_ASV_431 | Sputum | 0.010266 |
| Porphyromonas_ASV_1185 | Sputum | 0.033698 |
| Kingella_ASV_3691 | Sputum | 0.048782 |
| Streptococcus_ASV_443 | Sputum | 0.007367 |
| Capnocytophaga_ASV_734 | Sputum | 0.033775 |
| Veillonella_ASV_3148 | Sputum | 0.031327 |
| Porphyromonas_ASV_1190 | Sputum | 0.045202 |
| Oribacterium_ASV_2023 | Sputum | 0.045142 |
| Streptococcus_ASV_449 | Sputum | 0.006726 |
| Neisseria_ASV_3651 | Sputum | 0.015145 |
| Streptococcus_ASV_416 | Sputum | 0.022014 |
| Prevotella_ASV_1007 | Sputum | 0.033775 |
| Capnocytophaga_ASV_1412 | Sputum | 0.015432 |
| Leptotrichia_ASV_2792 | Sputum | 0.015395 |
| Stomatobaculum_ASV_2014 | Sputum | 0.033467 |
| Prevotella_ASV_1097 | Sputum | 0.013199 |
| Porphyromonas_ASV_1177 | Sputum | 0.015432 |
| Streptococcus_ASV_435 | Sputum | 0.015284 |
| Veillonella_ASV_3170 | Sputum | 0.006744 |
| Veillonella_ASV_3133 | Sputum | 0.00307 |
| Atopobium_ASV_3328 | Sputum | 0.033621 |
| Fusobacterium_ASV_2381 | Sputum | 0.014406 |
| Prevotella_ASV_1274 | Sputum | 0.015432 |
| Streptococcus_ASV_436 | Sputum | 0.033557 |
| Lautropia_ASV_3442 | Sputum | 0.017459 |
| Capnocytophaga_ASV_1411 | Sputum | 0.033698 |
| Streptococcus_ASV_544 | Sputum | 0.001104 |
| Streptococcus_ASV_432 | Sputum | 0.001107 |
| Porphyromonas_ASV_1187 | Sputum | 0.01296 |
| Cardiobacterium_ASV_2895 | Sputum | 0.015395 |
| Campylobacter_ASV_95 | Sputum | 0.000937 |
| Streptococcus_ASV_548 | Sputum | 0.005699 |
| Prevotella_ASV_928 | Sputum | 0.033698 |
| Haemophilus_ASV_2203 | Sputum | 0.012834 |
| Veillonella_ASV_3196 | Sputum | 0.003737 |
| Streptococcus_ASV_509 | Sputum | 0.000141 |
| Streptococcus_ASV_469. | Sputum | 0.002811 |
| Lautropia_ASV_3450 | Sputum | 0.01869 |
| Veillonella_ASV_3178 | Sputum | 0.002735 |
| Prevotella_ASV_924 | Sputum | 0.000193 |
| Streptococcus_ASV_415 | Sputum | 0.015315 |
| Capnocytophaga_ASV_1398 | Sputum | 0.033698 |
| Porphyromonas_ASV_1180 | Sputum | 0.019157 |
| Haemophilus_ASV_2180 | Sputum | 0.006744 |
| Prevotella_ASV_1116 | Sputum | 0.006744 |
| Neisseria_ASV_3692 | Sputum | 0.015284 |
| Prevotella_ASV_1110 | Sputum | 0.003729 |
| Streptococcus_ASV_521 | Sputum | 0.017846 |
| Streptococcus_ASV_461 | Sputum | 0.006726 |
| Neisseria_ASV_3627 | Sputum | 0.011092 |
| Prevotella_ASV_1060 | Sputum | 0.033698 |
| Capnocytophaga_ASV_1400 | Sputum | 0.033775 |
| Streptococcus_ASV_450 | Sputum | 0.003359 |
| Porphyromonas_ASV_1282 | Sputum | 0.015395 |
| Fusobacterium_ASV_2399 | Sputum | 0.015432 |
| Veillonella_ASV_3156 | Sputum | 0.003058 |
| Streptococcus_ASV_505 | Sputum | 0.038654 |
| Veillonella_ASV_3191 | Sputum | 0.033775 |
| Porphyromonas_ASV_1199 | Sputum | 0.015432 |
| Streptococcus_ASV_504 | Sputum | 0.023215 |

**Table S8: DESEq2 output for paired before treatment samples**

| Taxa | baseMean | log2FoldChange | lfcSE | stat | pvalue | padj |
| --- | --- | --- | --- | --- | --- | --- |
| Moraxella_ASV_2903 | 7.209665 | -3.56471 | 0.836973 | -4.25905 | 2.05E-05 | 0.002546 |
| Staphylococcus_ASV_339 | 3.424511 | 2.576535 | 0.774343 | 3.327382 | 0.000877 | 0.023113 |
| Corynebacterium_ASV_1841 | 25.82289 | -3.00432 | 0.893632 | -3.36193 | 0.000774 | 0.023113 |
| Corynebacterium_ASV_1843 | 3.555116 | 2.52358 | 0.737163 | 3.423368 | 0.000619 | 0.023113 |
| Neisseria_ASV_3681 | 4.579686 | 2.553966 | 0.771522 | 3.310294 | 0.000932 | 0.023113 |
| Streptococcus_ASV_415 | 5.19538 | -1.92015 | 0.658311 | -2.91679 | 0.003537 | 0.043853 |
| Ornithobacterium_ASV_702 | 2.787584 | -2.22157 | 0.735138 | -3.02198 | 0.002511 | 0.043853 |
| Haemophilus_ASV_2175 | 3.181487 | -1.8225 | 0.624264 | -2.91943 | 0.003507 | 0.043853 |
| Haemophilus_ASV_2272 | 4.756797 | 2.150555 | 0.734914 | 2.926266 | 0.003431 | 0.043853 |
| Veillonella_ASV_3128 | 3.573013 | -2.03292 | 0.668299 | -3.04193 | 0.002351 | 0.043853 |
| Porphyromonas_ASV_1199 | 2.876047 | 1.830613 | 0.642329 | 2.84996 | 0.004372 | 0.04929 |

**Table S9: DESEq2 output for paired after treatment samples**

| TaxaName | baseMean | log2FoldChange | lfcSE | stat | pvalue | padj |
| --- | --- | --- | --- | --- | --- | --- |
| Neisseria_ASV_3627 | 52.79287 | 5.100054 | 0.992616 | 5.137996 | 2.78E-07 | 5.86E-05 |
| Veillonella_ASV_3133 | 9.881152 | 3.255925 | 0.798898 | 4.075521 | 4.59E-05 | 0.003229 |
| Veillonella_ASV_3156 | 69.94568 | 3.873054 | 0.931709 | 4.156933 | 3.23E-05 | 0.003229 |
| Dolosigranulum_ASV_193 | 18.49213 | 3.575422 | 0.942982 | 3.79161 | 0.00015 | 0.005698 |
| Streptococcus_ASV_521 | 6.783653 | 2.793027 | 0.724992 | 3.852491 | 0.000117 | 0.005698 |
| Veillonella_ASV_3196 | 14.86331 | 3.194281 | 0.846872 | 3.771858 | 0.000162 | 0.005698 |
| Staphylococcus_ASV_331 | 12.83951 | -3.19251 | 0.857935 | -3.72116 | 0.000198 | 0.005978 |
| Streptococcus_ASV_432 | 4.431063 | 2.432311 | 0.681038 | 3.571478 | 0.000355 | 0.008322 |
| Cutibacterium_ASV_1886 | 4.205838 | -2.67104 | 0.743254 | -3.5937 | 0.000326 | 0.008322 |
| Campylobacter_ASV_95 | 4.885859 | 2.237695 | 0.642403 | 3.483323 | 0.000495 | 0.010449 |
| Streptococcus_ASV_509 | 3.701889 | 2.225804 | 0.657829 | 3.383559 | 0.000716 | 0.013046 |
| Rothia_ASV_1730 | 3.77848 | 2.490259 | 0.73855 | 3.371821 | 0.000747 | 0.013046 |
| Veillonella_ASV_3178 | 9.787428 | 2.602944 | 0.776653 | 3.351488 | 0.000804 | 0.013046 |
| Moraxella_ASV_2643 | 3.59697 | 2.531421 | 0.767763 | 3.297137 | 0.000977 | 0.014721 |
| Veillonella_ASV_3170 | 3.77813 | 2.373117 | 0.747645 | 3.174123 | 0.001503 | 0.021141 |
| Hydrogenophilus_ASV_3365 | 3.428754 | -2.45174 | 0.781824 | -3.13592 | 0.001713 | 0.022592 |
| Prevotella_ASV_924 | 26.83812 | 2.779433 | 0.893411 | 3.111036 | 0.001864 | 0.02314 |
| Leptotrichia_ASV_2851 | 3.331533 | 2.402077 | 0.785822 | 3.056768 | 0.002237 | 0.026227 |
| Micrococcus_ASV_1766 | 2.720239 | -2.0493 | 0.695643 | -2.9459 | 0.00322 | 0.03576 |
| Lautropia_ASV_3442 | 32.36676 | 2.659839 | 0.921297 | 2.88706 | 0.003889 | 0.041025 |
| Streptococcus_ASV_450 | 5.449457 | 1.919544 | 0.67126 | 2.859615 | 0.004242 | 0.042618 |
| Haemophilus_ASV_2180 | 4.362166 | 2.122551 | 0.757948 | 2.800392 | 0.005104 | 0.048953 |
